# Antibody Class(es) Predictor for Epitopes (AbCPE): A Multi-Label Classification Algorithm

**DOI:** 10.3389/fbinf.2021.709951

**Published:** 2021-09-07

**Authors:** Kiran Kadam, Noor Peerzada, Rajiv Karbhal, Sangeeta Sawant, Jayaraman Valadi, Urmila Kulkarni-Kale

**Affiliations:** ^1^ Bioinformatics Centre, Savitribai Phule Pune University, Pune, India; ^2^ Centre for Modeling and Simulation, Savitribai Phule Pune University, Pune, India; ^3^ Department of Computer Science, FLAME University, Pune, India

**Keywords:** epitope prediction, antibody, antibody class, multi-specificity, multi-label classification, antigen-antibody interaction, immunoinformatics, bioinformatics

## Abstract

Development of vaccines and therapeutic antibodies to deal with infectious and other diseases are the most perceptible scientific interventions that have had huge impact on public health including that in the current Covid-19 pandemic. From inactivation methodologies to reverse vaccinology, vaccine development strategies of 21st century have undergone several transformations and are moving towards rational design approaches. These developments are driven by data as the combinatorials involved in antigenic diversity of pathogens and immune repertoire of hosts are enormous. The computational prediction of epitopes is central to these developments and numerous B-cell epitope prediction methods developed over the years in the field of immunoinformatics have contributed enormously. Most of these methods predict epitopes that could potentially bind to an antibody regardless of its type and only a few account for antibody class specific epitope prediction. Recent studies have provided evidence of more than one class of antibodies being associated with a particular disease. Therefore, it is desirable to predict and prioritize ‘peptidome’ representing B-cell epitopes that can potentially bind to multiple classes of antibodies, as an open problem in immunoinformatics. To address this, AbCPE, a novel algorithm based on multi-label classification approach has been developed for prediction of antibody class(es) to which an epitope can potentially bind. The epitopes binding to one or more antibody classes (IgG, IgE, IgA and IgM) have been used as a knowledgebase to derive features for prediction. Multi-label algorithms, Binary Relevance and Label Powerset were applied along with Random Forest and AdaBoost. Classifier performance was assessed using evaluation measures like Hamming Loss, Precision, Recall and F1 score. The Binary Relevance model based on dipeptide composition, Random Forest and AdaBoost achieved the best results with Hamming Loss of 0.1121 and 0.1074 on training and test sets respectively. The results obtained by AbCPE are promising. To the best of our knowledge, this is the first multi-label method developed for prediction of antibody class(es) for sequential B-cell epitopes and is expected to bring a paradigm shift in the field of immunoinformatics and immunotherapeutic developments in synthetic biology. The AbCPE web server is available at http://bioinfo.unipune.ac.in/AbCPE/Home.html.

## Introduction

Antibody-mediated immune response is characterized by generation of antibodies (immunoglobulins) from activated B-cells which are targeted at specific pathogens or pathogenic molecules (antigens). Antigen-antibody interactions are fundamental to adaptive immunity and in recent years mapping of these interactions has gained tremendous significance in the field of immunology (Abbott et al., 2014). An epitope is an immunogenic region of an antigen which is specifically recognized by or interacts with antibodies/specialized lymphocytes. An antibody-binding epitope, also known as B-cell epitope can either be linear (sequential) or conformational (discontinuous) in nature. A linear epitope consists of a contiguous stretch of amino acids while conformational epitope comprises of one or more linear epitopes and a few amino acids located at different positions in the antigen sequence that lie in close proximity within the folded protein (Kolaskar and Kulkarni-Kale, 1999; Kulkarni-Kale et al., 2005).

Identification and characterization of epitopes is considered to be of paramount importance because of their applications in various areas like therapeutics (Wilson and Andrews, 2012), diagnostics (Ahmad et al., 2016) and peptide-based vaccines (Dudek et al., 2010; Ahmad et al., 2016). Over the years, a large number of B-cell epitopes have been characterized using experimental approaches. The Immune Epitope Database (IEDB) archives data on epitopes derived from diverse sources of antigens and emerged as a primary repository of epitope data (Vita et al., 2019). The computational methods complement experimental approaches by not only reducing the search space, time and costs but also accelerate the pace of discovery of epitopes by bringing in the power of data and data analytics (Abbott et al., 2014). Therefore, computational prediction of B-cell epitopes has emerged as a very effective alternative for large scale characterization of epitopes (Potocnakova et al., 2016). Many computational methods have been developed for prediction of linear as well as conformational epitopes (Yao et al., 2013; Sanchez-Trincado et al., 2017) and some of these have also been made available on IEDB portal. Numerous epitope prediction methods based on machine learning algorithms have been developed recently, which utilize variety of features derived from sequences and/or structures. These include linear and conformational epitope prediction methods such as LBtope (Singh et al., 2013), CBTOPE (Ansari and Raghava, 2010), iBCE-EL (Manavalan et al., 2018), iLBE (Hasan et al., 2020) as well as a method that deals with prediction of antibody specific B-cell epitopes (Jespersen et al., 2019).

Epitopes are recognized by antibodies and are critical constituent of antigen-antibody reactions. Antibodies are attributed to be responsible for the specificity in an antigen-antibody reaction which is mediated through paratopes, which are complementary to epitopes. The paratopes are presented at the interface of the complementarity determining regions (CDRs) on both, light and heavy chains of an antibody. In general, antibodies are also involved in variety of important functions associated with the immune system such as compliment activation, mast cell binding, cell-mediated cytotoxicity, phagocytosis, hypersensitivity etc. (Galli and Tsai, 2012; Forthal and Finzi 2018; Tay et al., 2019; Goldberg and Ackerman, 2020). Based on the type of heavy chain present, antibodies are broadly divided into five classes viz. Immunoglobulin G (IgG), Immunoglobulin E (IgE), Immunoglobulin M (IgM), Immunoglobulin A (IgA) and Immunoglobulin D (IgD). Each of these classes is associated with specific effector function/s. For instance, IgG is the most predominant immunoglobulin in serum that binds to varied types of antigens and its four subclasses are responsible for different effector functions (Vidarsson et al., 2014). IgE represents the key antibody associated with mediation of allergic reactions and plays a central role in allergic diseases like allergic asthma, allergic rhinitis, and food allergy (Platts-Mills, 2001). IgA is an important serum immunoglobulin, apart from being a major antibody present in secretions. It is the principal mucosal antibody class, responsible for neutralization of variety of pathogenic microbes including viruses (Woof and Kerr, 2006). IgM is the first class of antibodies produced during a primary antibody response which also plays a crucial immunological role in inflammation and autoimmunity (Grönwall and Silverman, 2014). IgD denotes an ancestral class of antibody which is produced as membrane-bound as well as a secreted antibody (Chen and Cerutti, 2011). Although the biology and function of IgD had remained poorly understood till recent years, latest research has helped to elucidate its role in the regulation of tolerogenic and protective B cell responses, mucosal immunity and as a transmembrane receptor (Gutzeit et al., 2018).

Inferences drawn from the past studies have indicated that a particular pathogen/antigen is responsible for induction of specific class/subclass of antibodies. For instance, IgG and its subclasses are found to be specifically associated with protozoans infections (Garraud et al., 2003; Flueck et al., 2009), autoimmune diseases (Zhang et al., 2015) and filarial infections (Ottesen et al., 1985). Despite possessing a broad range of functions, IgG antibodies also represent the most potent class of antibodies for designing therapeutic monoclonal antibodies for several infectious diseases (Irani et al., 2015). IgA is known to be mainly associated with inhibition of pathogen attachment to mucosal surfaces by interacting with specific receptors (Jain and Rosenthal, 2011). It is also the main class of antibody produced in case of viral infections (Blutt and Conner, 2013). IgM is shown to be specifically linked with regulation of immune responses, protection from autoimmune diseases and recognition and clearance of apoptotic cells (Peng et al., 2005; Grönwall et al., 2012). As mentioned earlier, IgE is specifically responsible for induction of IgE-mediated allergic responses (Platts-Mills, 2001).

In certain cases, however, it has been observed that more than one class of antibodies are associated with pathophysiology of a few diseases. For example, IgG and IgA have been shown to play important roles in number of allergic diseases (Gloudemans et al., 2013; Scott-Taylor et al., 2018). IgG antibodies have potential to act as blocking antibodies in allergic reactions and hence play a critical role in design of allergen specific immunotherapy (Aalberse, 2011). Some studies have revealed that certain Dengue virus antigens specifically interact with both IgM and IgG antibodies and this can be utilized for efficient diagnosis of the disease (Hapugoda et al., 2007; Lee et al., 2015). The immune response in patients affected by recent outbreak of 2019 novel coronavirus (SARS-CoV-2) is shown to be comprising of IgG, IgM and IgA antibodies and serological tests based on detection of these antibodies have shown immense potential in diagnosis of the disease (Ma et al., 2020; Chen et al., 2021).

Thus, it is evident that study of epitope repertoire of specific class/classes of antibodies represents a very relevant problem in immunoinformatics. However, very few computational methods have been developed till date which deal with prediction of epitopes binding to specific class of antibodies. These mainly include methods that deal with prediction of IgE-binding epitopes in case of allergens and web-servers like AlgPred 2.0 (Sharma et al., 2020), SPADE (Dall’Antonia and Keller, 2019) and a standalone tool BCIgPRED (Saravanan and Gautham, 2018) are examples of such methods. Raghava and co-workers have studied the problem of prediction of epitopes that can induce a specific class of antibody and developed a web-server IgPred for prediction of IgG, IgE and IgA binding epitopes (Gupta et al., 2013).

Thus, prediction of epitopes binding with specific and/or multiple classes of antibodies needs to be addressed as a multi-label classification problem comprising of instances that are simultaneously associated with more than one class (label). Recent years have witnessed considerable increase in the use of multi-label classification methods in the area of bioinformatics. It has been applied in protein subcellular localization prediction (Xiao et al., 2011; Lin et al., 2013), protein subchloroplast localization prediction (Wang et al., 2015), prediction of membrane protein types in animals (Zou, 2014), detection of multi-functional enzyme (Che et al., 2016), identification of phosphorylated proteins in humans (Qiu et al., 2017) etc.

Though B-cell epitope prediction algorithms have been a very important part of immunoinformatics since long time and they have performed very efficiently in their objective, none of these methods, except for IgPred (Gupta et al., 2013), addresses prediction of epitopes specific for a single type of antibody, leave alone dealing with multispecificity. These classical methods are based on the traditionally accepted notion of absolute antibody specificity which denotes that an antibody is highly specific for a single antigen/epitope. In addition, polyspecificity or multispecificity of antibodies has also emerged as a prominent phenomenon in recent times which could provide explanation for significant variability observed in terms of antigen/epitope recognition (Van Regenmortel, 2014), illustrating how an antibody interacts with multiple epitopes while mediating specificity in recognition of every individual epitope. The IgPred method is useful for epitope prediction for specific class of antibody but none of the methods available till date account for epitopes binding to multiple classes of antibodies by using a multi-label approach. Thus, to address a complex biological problem like prediction of epitopes capable of being recognized by and potentially bind to one or more classes of antibodies will require a novel approach which has not been applied for epitope predictions. This manuscript, therefore documents our attempt to address epitope prediction problem by formulating it as a multi-label classification framework. The study employs antibody class-specific epitope data compiled from IEDB, sequence based features and multi-label classification algorithms such as Binary Relevance and Label Powerset to build models.

## Materials and Methods

### Datasets

#### Dataset for Model Building and Evaluation

The dataset used in this study is compiled from the Immune Epitope Database (IEDB) (Vita et al., 2019) by taking into account linear (sequential) B-cell epitopes of length 5–50 amino acids from only positive B-cell assays. These epitopes belong to all types of pathogens such as bacteria, viruses, fungi in addition to allergens and epitopes associated with autoimmunity. All the epitopes from various sources are collated together and used as a single dataset to eliminate any influence of host specific codon usage/amino acid preferences.

Epitope sequences specific for four antibody heavy chain classes viz. IgG, IgE, IgA and IgM are extracted and divided into four labels, one for each heavy chain class. IgD antibody class is not considered due to the lack of data on epitopes that can bind IgD. Epitope sequences that are able to bind more than one class of antibody are also extracted and curated using specifically written Perl scripts. These epitopes denote multi-label instances as they bind to more than one class of antibody and are assigned appropriate labels. The final dataset comprises of a total of 10,744 epitope sequences belonging to 4 labels as listed in Table 1. Our goal was to design a model which predicts the correct antibody label for every epitope. Therefore, binary notation for each label was defined wherein each epitope is denoted in terms of 4 main labels (antibody classes) (Table 1). Thus, the targets here are decomposed into a set of four binary labels.

**TABLE 1:** Composition of datasets used in the study.

Antibody class/es	Binary notation	Epitope entries	
		Dataset	SARS-CoV-2
IgG	1 0 0 0	6,027	166
IgE	0 1 0 0	1,512	—
IgA	0 0 1 0	412	4
IgM	0 0 0 1	999	5
IgG + IgE	1 1 0 0	748	—
IgG + IgM	1 0 0 1	701	—
IgG + IgA	1 0 1 0	242	—
IgE + IgA	0 1 1 0	10	—
IgG + IgM + IgA	1 0 1 1	62	1
IgG + IgM + IgE	1 1 0 1	20	—
IgG + IgE + IgA	1 1 1 0	11	—

#### SARS-CoV-2 Dataset

A test dataset comprising of antibody binding epitopes from novel coronavirus, SARS-CoV-2 that is responsible for Covid-19 pandemic is also curated from the IEDB. The dataset contains total of 176 epitopes with humans as host organism, out of which 166 belong to the IgG binding class, 4 epitopes to IgA, 5 epitopes to IgM while 1 epitope binds to both IgG and IgM. The performance of AbCPE server is evaluated on this dataset.

### Sequence-Based Features

Extraction of relevant features from a protein/peptide sequence is a critical component of machine learning method development (Kadam et al., 2014). Sequence-based features used in this study are described briefly.

#### Amino Acid Composition

AAC represents the simplest feature which summarizes the global information of variable length protein/peptide sequence into a fixed length pattern. It is denoted by a feature vector of 20 dimensions in which fraction of standard twenty amino acids is represented. The frequency of all the 20 natural amino acids were calculated as:

$$F\left(i\right)=\frac{\mathrm{Ni}}{N}\;,i=\mathrm{1,2,3},\ldots \ldots \mathrm{..20}$$ (1)

where *F(i)* is the frequency of amino acid type *i* and *N* is the length of the peptide sequence.

#### Dipeptide Composition

The most important benefit of using DC is the inclusion of sequence-order information of the protein/peptide, which gets omitted in case of AAC. DC captures frequencies of every two consecutive amino acid residues in a variable length protein/peptide sequence. DC is denoted by a feature vector of 400 dimensions and calculated as:

$$F\left(i,j\right)=\frac{\mathrm{Nij}}{N-1}\;,i,j=\mathrm{1,2,3},\ldots \ldots \mathrm{..20}$$ (2)

where *F(i,j)* is the frequency of dipeptide formed by amino acid types *i* and *j* while *N* is the length of the peptide sequence.

#### Pseudo Amino Acid Composition

PseAAC is a feature encoding method proposed by (Chou, 2001) with the objective of including the sequence-order information in sequential representation of protein samples. Both Type 1 and Type 2 pseudo amino acid composition (Shen and Chou, 2008) are used to build the prediction models.

##### Type 1 Pseudo Amino Acid Composition

It is very commonly used PseAAC which is also known as the parallel-correlation type pseudo amino acid composition (Chou, 2001). PseAAC1 generates a set of 20+λ discrete numbers to denote a protein where first 20 descriptors represent the AAC and the additional ones represent the sequence-order information.

##### Type 2 Pseudo Amino Acid Composition

It is also known as amphiphilic or series-correlation type pseudo amino acid composition in which more importance is given to the distribution of the hydrophobic and hydrophilic residues (Chou, 2005). It represents a protein by 20 + i∗λ descriptors where first 20 descriptors represent common AAC and *i* denotes the number of amino acid attributes chosen while calculating PseAAC2.

#### Combined Feature Set

Prediction models were also developed using a collective feature set prepared from four different types of sequence-based features. This was accomplished by combining AAC, DC, PseAAC1 and PseAAC2 features.

### Evaluation of Features

In order to choose a subset of informative features from the given feature set, feature ranking protocol was performed on all the four feature sets using Waikato Environment for Knowledge Analysis (WEKA) software (Hall et al., 2009). Information Gain (InfoGain), a filter based feature selection technique is employed for attribute evaluation which measures the value of a feature by calculating information gain with respect to the class and assigning it a specific rank (Saeys et al., 2007).

### Prediction Algorithm

**FIGURE 1:**
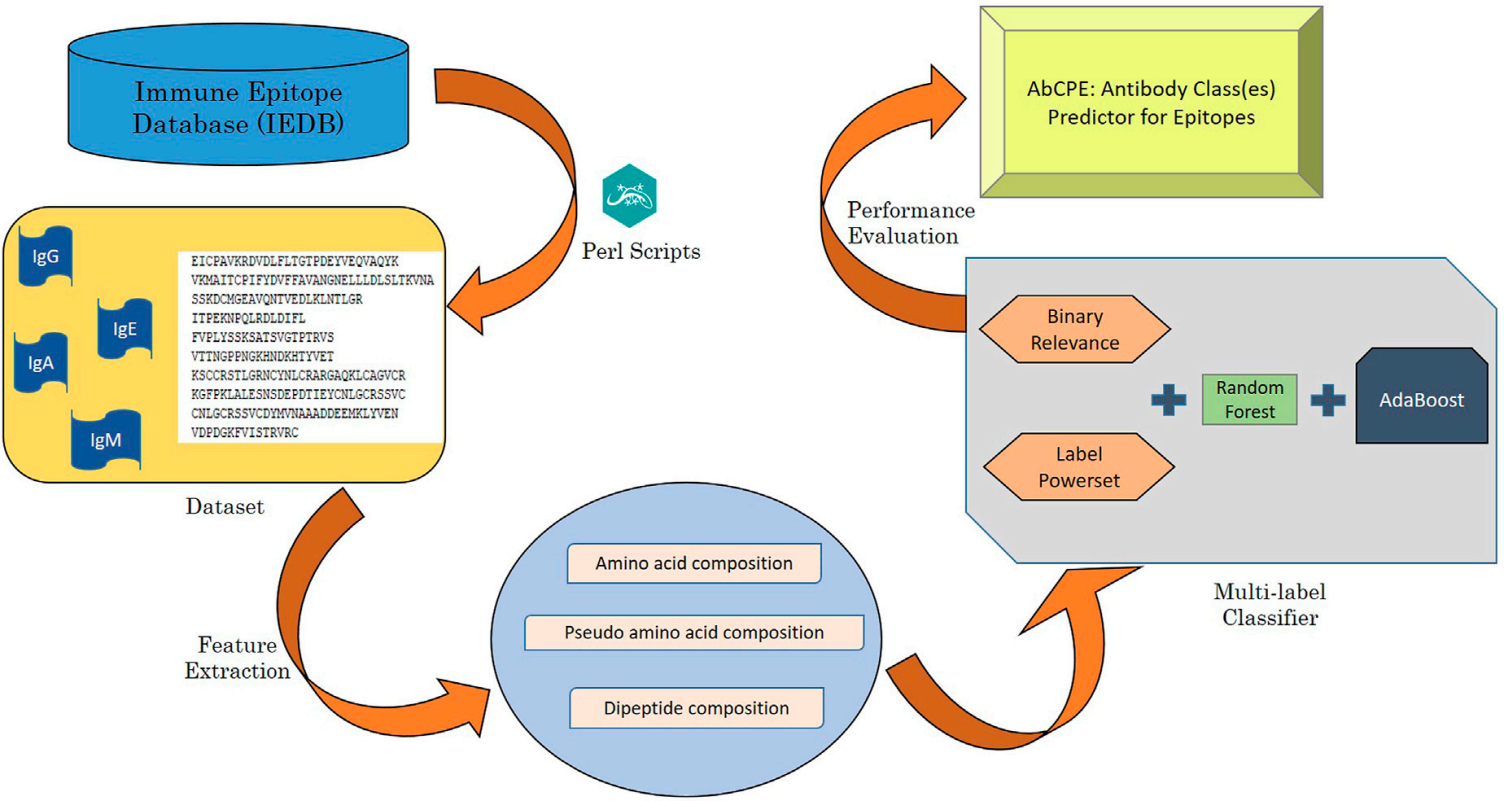
Diagrammatic illustration of AbCPE algorithm.

The algorithm for prediction of antibody class specific B-cell epitopes has been designed using a multi-label classification problem and is depicted in Figure 1. Multi-label dataset has been handled by transforming multi-label problem into a single label problem by employing two problem transformation methods followed by application of traditional classification algorithm.

#### Binary Relevance

It is one of the most commonly used problem transformation methods wherein the multi-label classification problem is converted into single label classification problems (Tsoumakas and Katakis, 2007). The transformation is achieved by converting multi-label problem into k binary classification problems (where, k = |L|, total number of classes). In this epitope prediction problem, four binary classification problems needed to be solved which denote four antibody classes. In the first classifier, examples showing IgG binding (irrespective of presence or absence of epitopes with other activities) are considered positive and examples not showing IgG binding are treated as negative. Likewise three more classifiers are considered for remaining three antibody labels. After creating four binary classification models, test examples are sent through each of the classifiers for identification of presence or absence of a label (activity).

#### Label Powerset

In this approach, instances belonging to each combination of label(s) is considered as a separate class (Tsoumakas and Vlahavas, 2007). For example, epitopes binding to only IgG are classified as class 1; epitopes binding to IgE, IgA and IgM are grouped into class 2, 3 and 4 respectively. Epitopes binding to both IgG and IgE are classified as class 5. Epitopes binding to IgG and IgM, IgG and IgA, IgE and IgA are classified as class 6, 7, and 8 respectively. Thus the dataset is converted into 15 distinct classes.

#### Base Classifier

In the current work, Random Forest (RF) is employed as the base classifier (Breiman, 2001) as it is one of the most commonly applied machine learning methods in bioinformatics. It is a type of ensemble classifier which denotes an improvement over bagged decision trees. The output of RF classifier is a linear combination of input features which are mapped between 0 and 1 using a sigmoid function.

#### AdaBoost Classifier

Boosting is an ensemble based method used to improve classification problems by combining several weak classifiers, leading to development of strong classifiers. AdaBoost is one of the most popular ensemble learning methods first introduced by Freund and Schapire (1997). It combines several basic and weak predictors together to produce better prediction accuracies. Starting with a weak classifier, AdaBoost iteratively evolves a strong learning algorithm, each time improving the predictive capability by adding another basic predictor (classifier) into the prediction task.

The skeleton of AdaBoost is depicted below:

a) Consider a training set
b) Initialize and normalize the weight D = (x1, y1), . . . , (x_m_, y_m_), . . . , y ∈ {−1, +1}
c) Repeat from t = 1,... T, executing the following sub steps. (i) Perform training on the training set with distribution D_t_, (ii) Get base classifier which results in the least error, (iii) Update the weight focused on incorrect samples and set the new weights
d) Output the final strong classifier H

Prediction performance of all the extracted features is also evaluated by using only the base classifier. In this case, only Random Forest is used to perform predictions without boosting it with AdaBoost. This approach will be helpful in assessing efficacy of the base classifier alone as well as the effect of boosting the classifier with AdaBoost.

### Performance Evaluation and Comparison

We evaluated the performance of every model by creating five different random splits of the entire data to ensure statistically unbiased estimation. The dataset is divided into five different 80:20 random splits by using five random seeds wherein 80 and 20% data are used for training and testing, respectively. Each of the five distinct 80% training splits are used for estimating cross-validation performance measures. The corresponding test performance measures are computed with the five 20% test splits. Finally, we computed the average of five different cross-validation and test performance measures. These evaluations are carried out by employing all the feature sets with both Binary Relevance and Label Powerset algorithms with only Random Forest as well as using Random Forest in combination with AdaBoost. Principal component analysis (PCA) plots are drawn on each of the five training and test sets obtained from 80:20 splits for the best performing feature set, to assess potential overlap between training and corresponding test splits.

### Performance Measures

Performance evaluation measures such as Hamming Loss, Precision, Recall and F1 score are used to assess the performance of multi-label models. Hamming Loss (HL) is an example-based evaluation metric. It denotes a loss function which calculates the proportion of misclassified labels to the total number of labels, averaged over all the samples (Schapire and Singer, 2000). The smaller the value of hamming loss, the better is the efficiency of classifier. Precision (P) represents the fraction of relevant instances among the retrieved instances while Recall (R) denotes the fraction of the total number of relevant instances that were actually retrieved. F1 score is the harmonic mean between Precision and Recall. In order to assess the label based performance of the classifier, both macro and micro averaged values of Precision, Recall and F1 score are considered.

These evaluation measure are calculated as follows. In the definitions, yi represents the set of true labels of example xi, while h(xi) denotes the set of predicted labels for same example. N denotes total number of examples and Q is total number of labels. For calculating macro and micro averaged Precision, Recall and F1 score, TP_j_, TN_j_, FP_j_ and FN_j_ denote true positives, true negatives, false positives and false negatives respectively, for the label λ_j_ considered as a binary class.

Hamming Loss is calculated as:

$$\mathrm{HL}=\frac{1}{N}\overset{N}{\underset{i=1}{\sum }}\frac{1}{Q}\left|h\left(\mathrm{xi}\right)\;\Delta \;\mathrm{yi}\right|$$ (3)

where Δ denotes symmetric difference between two sets.

Precision is calculated as:

$$P\_\mathrm{macro}=\frac{1}{Q}\overset{Q}{\underset{j=1}{\sum }}\frac{\mathrm{TPj}}{\mathrm{TPj}+\mathrm{FPj}}$$ (4)

$$P\_\mathrm{micro}=\frac{\sum_{j=1}^{Q}\mathrm{TPj}}{\sum_{j=1}^{Q}\mathrm{TPj}+\;\sum_{j=1}^{Q}\mathrm{FPj}}$$ (5)

Recall is calculated as:

$$R\_\mathrm{macro}=\frac{1}{Q}\overset{Q}{\underset{j=1}{\sum }}\frac{\mathrm{TPj}}{\mathrm{TPj}+\mathrm{FNj}}$$ (6)

$$R\_\mathrm{micro}=\frac{\sum_{j=1}^{Q}\mathrm{TPj}}{\sum_{j=1}^{Q}\mathrm{TPj}+\;\sum_{j=1}^{Q}\mathrm{FNj}}$$ (7)

F1 score is calculated as:

$$F1\_\mathrm{macro}=\frac{1}{Q}\overset{Q}{\underset{j=1}{\sum }}\frac{2*\mathrm{Pj}*\mathrm{Rj}}{\mathrm{Pj}+\mathrm{Rj}}$$ (8)

where P_j_ and R_j_ are the Precision and Recall for all λ_j_ ∈ h(xi) from λ_j_ ∈ yi.

$$F1\_\mathrm{micro}=\frac{2*P\_\mathrm{micro}*R\_\mathrm{micro}}{P\_\mathrm{micro}+R\_\mathrm{micro}}$$ (9)

### Scripts and Software

Web server is built using Apache HTTP Server (Version-2.2.21). The web interface is developed using HTML, CSS and JavaScript. Features are calculated using in-house developed Perl (Version 5.24.1) scripts. Python (Version 3.6) is used to write scripts for the AdaBoost classifier. Various Python packages like Pandas, NumPy, Pickle are also employed to code the algorithm.

## Results

The performance and relative contributions of the sequence-based features such as amino acid composition, dipeptide composition and PseAAC that are employed to develop the algorithm are evaluated and summarized.

### Percent Amino Acid Composition

**FIGURE 2:**
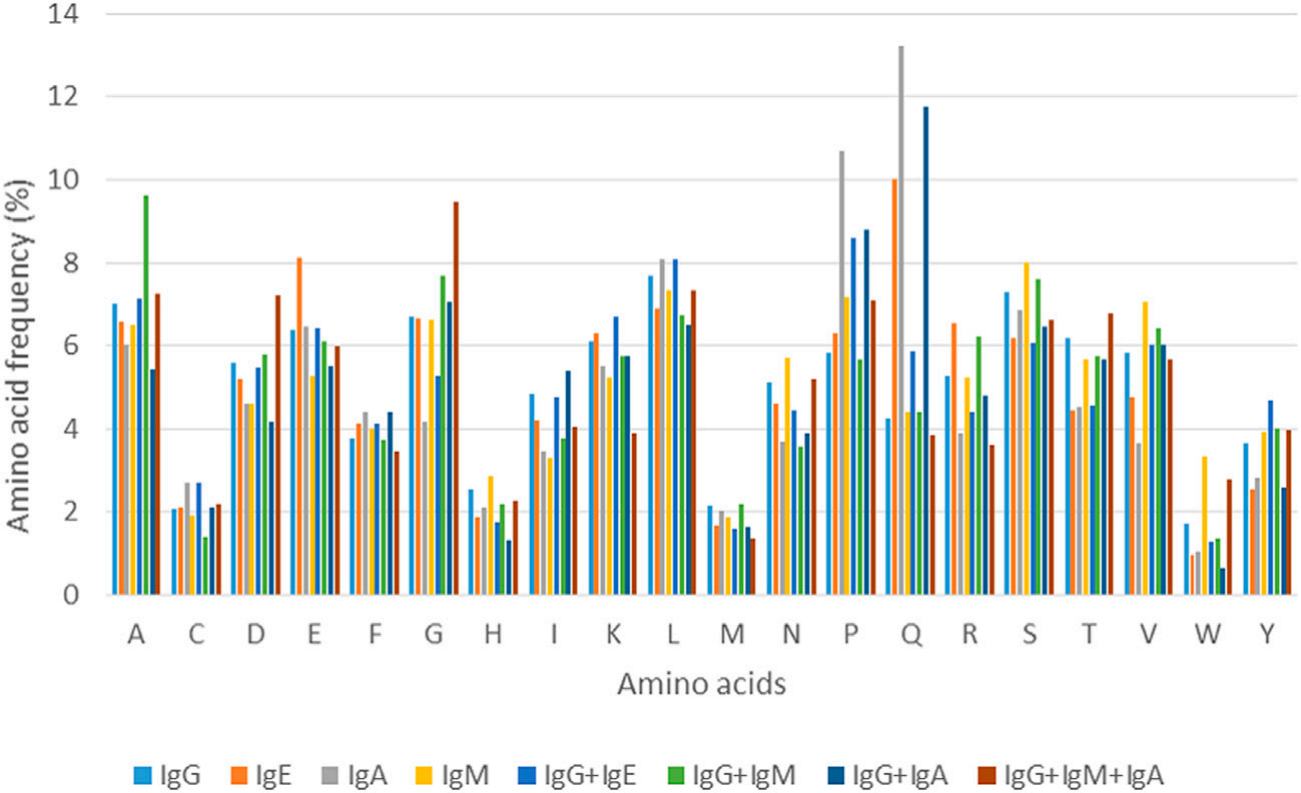
Distribution of amino acids in various epitope classes.

Analysis of amino acid composition of each epitope class binding to a specific class/classes of antibody/ies provided some important insights into amino acids that constitute these epitopes. Figure 2 shows the variation in amino acid composition for each class of epitope, represented by percent amino acid composition (Three antibody classes are excluded due to lack of sufficient examples).

For the four epitopes classes involved in binding to a single class of antibody, it can be seen that the amino acid frequencies vary to some degree for majority of residues. However, as can be seen in Figure 2, considerable difference can be observed in proportions of few residues such as glutamic acid (E) and glutamine (Q) in IgE and IgA binding epitopes. For epitope classes that bind to more than one class of antibody, differences in percent amino acid composition can be seen for most of the residues with some explicit trends, like for amino acids glutamine (Q), proline (P) and valine (V) that are more predominantly present while histidine (H) and cysteine (C) are less common. These observations indicate that almost all the 20 amino acids show variation in their occurrence in epitopes binding to specific antibody class/es. This variation in amino acid composition can therefore be utilized in terms of compositional features to design and develop the models and the algorithms for prediction of epitopes that bind to specific antibody class/es.

### Feature Evaluation by Waikato Environment for Knowledge Analysis-Information Gain

Assessment of the features in all four feature sets by Information Gain attribute evaluator provides interesting results. Based on InfoGain feature rankings, the most relevant features in each dataset are obtained. For AAC feature set, glutamine (Q) is found to be the most valuable amino acid, followed by leucine (L), serine (S), and glycine (G). In addition to these residues, alanine (A), glutamic acid (E), valine (V) and tyrosine (Y) are also observed as important amino acids. In the DC dataset, the glutamine-glutamine (Q-Q) dipeptide is ranked the highest implying its significance in DC based classifiers. Apart from Q-Q, proline-glutamine (P-Q), glutamine-proline (Q-P), phenylalanine-proline (F-P), proline-tyrosine (P-Y) and glutamine-phenylalanine (Q-F) are the other dipeptides with maximum information content in relation to all the antibody classes. For both PseAAC1 and PseAAC2 feature sets, the attribute denoting glutamine (Q) residue is found to be the most informative one. Features which represent tyrosine (Y), valine (V) and arginine (R) are the other significant attributes for these two datasets.

### Evaluation of Performance

#### Training Base Classifier (Random Forest) Without AdaBoost

Random Forest is used as a base classifier in the current prediction methodology, for both BR and LP algorithms. Predictions are performed without using AdaBoost, employing only the base classifier on all the feature sets. Both BR and LP algorithms are evaluated by the method explained in *Performance Evaluation and Comparison*. The average 5-fold cross-validation performance estimates in terms of Hamming Loss and other measures are shown in Tables 2, 3.

**TABLE 2:** Training performance outcome for BR-RF classifier (Note that the best performing feature set is shown in bold).

Feature set	HL	Micro average			Macro average		
		P	R	F1	P	R	F1
AAC	0.1465	0.8076	0.6586	0.7255	0.7975	0.4222	0.5086
**DC**	**0.1392**	**0.8165**	**0.6791**	**0.7415**	**0.8192**	**0.4490**	**0.5392**
PseAAC1	0.1594	0.7884	0.6258	0.6977	0.7492	0.3689	0.4409
PseAAC2	0.1629	0.7834	0.6165	0.6899	0.7405	0.3586	0.4283
Combined feature set	0.1539	0.7987	0.6370	0.7088	0.7975	0.3809	0.4582

**TABLE 3:** Training performance outcome for LP-RF classifier (Note that the best performing feature set is shown in bold).

Feature set	HL	Micro average			Macro average		
		P	R	F1	P	R	F1
AAC	0.1482	0.7744	0.6997	0.7352	0.7044	0.4699	0.5360
**DC**	**0.1373**	**0.7915**	**0.7237**	**0.7561**	**0.7528**	**0.5007**	**0.5731**
PseAAC1	0.1571	0.7599	0.6805	0.7180	0.6811	0.4384	0.5005
PseAAC2	0.1631	0.7502	0.6672	0.7062	0.6627	0.4156	0.4740
Combined feature set	0.1530	0.7688	0.6871	0.7248	0.7068	0.4385	0.5040

Both BR (Table 2) and LP (Table 3) algorithms are observed to perform very efficiently when employed with the base classifier Random Forest. Based on the Hamming Loss and other measures, it is observed that the LP-Random Forest classifier performs marginally better than the BR-Random Forest classifier for four individual feature sets as well as for the combined feature set. DC is found to be the best performing feature set for both methods (as shown in bold in Table 2 and Table 3) although efficiency of amino acid composition is also very good.

#### Training Base Classifier (Random Forest) With AdaBoost

All four types of features extracted from epitope sequences in the dataset and the combined feature set obtained by considering all four types of features are subjected to 5-fold cross-validation and testing procedures as explained in *Performance Evaluation and Comparison* by employing the base classifier (Random Forest) along with AdaBoost. For both BR and LP algorithms, performance is evaluated using Hamming Loss as well as macro and micro averaged values of Precision, Recall and F1 score.

Based on the average values of all the performance measures, it is seen that both BR-RF-AdaBoost classifier (Table 4) and LP-RF-AdaBoost classifier (Table 5) provide very good prediction results, with BR-RF-AdaBoost classifier offering relatively superior prediction performance than the LP-RF-AdaBoost classifier. The BR-RF-AdaBoost model is observed to give better results for all feature sets as compared to the LP-AdaBoost classifier, denoted by better Hamming Loss and other measures. The dipeptide feature set in combination with BR, base classifier and AdaBoost is shown to be the best model with Hamming Loss of 0.1121.

**TABLE 4:** Training performance outcome for BR-RF-AdaBoost classifier (Note that the best performing feature set is shown in bold).

Feature set	HL	Micro average			Macro average		
		P	R	F1	P	R	F1
AAC	0.1259	0.8197	0.7331	0.7740	0.8474	0.4904	0.5766
**DC**	**0.1121**	**0.8365**	**0.7688**	**0.8012**	**0.8281**	**0.5700**	**0.6521**
PseAAC1	0.1416	0.7971	0.6954	0.7427	0.8453	0.4156	0.4876
PseAAC2	0.1518	0.7806	0.6725	0.7225	0.8470	0.3726	0.4283
Combined feature set	0.1305	0.8093	0.7274	0.7662	0.8575	0.4682	0.5504

**TABLE 5:** Training performance outcome for LP-RF-AdaBoost classifier (Note that the best performing feature set is shown in bold).

Feature set	HL	Micro average			Macro average		
		P	R	F1	P	R	F1
AAC	0.1304	0.8088	0.7285	0.7666	0.8277	0.4844	0.5670
**DC**	**0.1169**	**0.8255**	**0.7637**	**0.7934**	**0.8475**	**0.5424**	**0.6287**
PseAAC1	0.1456	0.7867	0.6926	0.7366	0.8229	0.4143	0.4828
PseAAC2	0.1567	0.7688	0.6677	0.7147	0.8251	0.3620	0.4097
Combined feature set	0.1468	0.7850	0.6895	0.7342	0.8542	0.4053	0.4719

Hamming Loss and both micro and macro averaged measures obtained in 5-fold cross-validation clearly indicate that the overall prediction performance of both the algorithms is very efficient for multi-label prediction of epitopes. AdaBoost in combination with both BR or LP algorithm and Random Forest gives considerably better results than the combination of BR or LP algorithm and Random Forest alone. Thus, it is established that employing AdaBoost enhances the prediction efficiency of both BR and LP classifiers. It is observed that all five feature sets perform quite well on both the classifiers, although DC based models provide the best results (as shown in bold in Table 4 and Table 5) followed closely by AAC feature set. The dipeptide model derived from BR, RF, and AdaBoost is found to be the most efficient one, with Hamming Loss of 0.1121.

### Predictions on Test Sets

The corresponding average test measures obtained using average of Hamming Loss in addition to micro and macro averaged Precision, Recall and F1 scores for different models are shown in Table 6. Both Binary Relevance and Label Powerset based models perform very efficiently on test sets, with former giving superior results than the latter. These include models that utilize AdaBoost as well as models that employ only the base classifier (Random Forest). AdaBoost based models are observed to provide better performance than models employing only Random Forest. For both BR and LP classifiers, the DC, AAC and the combined feature set are found to be the best performing feature sets. The best prediction performance is obtained from the model based on DC in combination with BR, Random Forest and AdaBoost with average Hamming Loss of 0.1074 and very good precision, recall and F1 score values, as displayed in bold in Table 6. Like the results of training, the model based on the AAC feature set in combination with BR, Random Forest and AdaBoost provides the second best performance on the test sets with average Hamming Loss of 0.1224.

**TABLE 6:** Average prediction performance for test sets (Note that the best performing model is shown in bold).

Feature set	Classifier	HL	Micro average			Macro average		
			P	R	F1	P	R	F1
AAC	BR-RF-AdaBoost	0.1224	0.8247	0.7410	0.7806	0.8492	0.5020	0.5898
	BR-RF	0.1433	0.8130	0.6650	0.7316	0.8078	0.4260	0.5133
	LP-RF-AdaBoost	0.1271	0.8125	0.7377	0.7733	0.8351	0.5017	0.5859
	LP-RF	0.1458	0.7772	0.7061	0.7399	0.7207	0.4845	0.5531
**DC**	**BR-RF-AdaBoost**	**0.1074**	**0.8418**	**0.7813**	**0.8104**	**0.8236**	**0.5926**	**0.6708**
	BR-RF	0.1370	0.8191	0.6848	0.7459	0.8083	0.4615	0.5525
	LP-RF-AdaBoost	0.1137	0.8283	0.7732	0.7998	0.8421	0.5611	0.6454
	LP-RF	0.1335	0.7961	0.7334	0.7635	0.7563	0.5167	0.5888
PseAAC1	BR-RF-AdaBoost	0.1395	0.7990	0.7016	0.7472	0.8503	0.4247	0.4996
	BR-RF	0.1578	0.7909	0.6293	0.7009	0.7747	0.3746	0.4498
	LP-RF-AdaBoost	0.1450	0.7870	0.6943	0.7378	0.8319	0.4169	0.4866
	LP-RF	0.1551	0.7633	0.6839	0.7214	0.6936	0.4409	0.5049
PseAAC2	BR-RF-AdaBoost	0.1513	0.7792	0.6765	0.7242	0.8435	0.3816	0.4405
	BR-RF	0.1599	0.7903	0.6231	0.6960	0.7480	0.3648	0.4358
	LP-RF-AdaBoost	0.1540	0.7727	0.6740	0.7200	0.8461	0.3737	0.4274
	LP-RF	0.1618	0.7515	0.6709	0.7089	0.6679	0.4222	0.4817
Combined feature set	BR-RF-AdaBoost	0.1271	0.8133	0.7363	0.7729	0.8605	0.4837	0.5685
	BR-RF	0.1516	0.8031	0.6410	0.7129	0.8039	0.3847	0.4635
	LP-RF-AdaBoost	0.1452	0.7863	0.6947	0.7376	0.8552	0.4177	0.4879
	LP-RF	0.1491	0.7741	0.6954	0.7326	0.7304	0.4536	0.5228

Principal component analysis (PCA) plots drawn on each of the five training and test splits for the dipeptide composition feature set are provided in the Supplementary Material (Supplementary Figures S1–S5). Based on the PCA plots, considerable separation between corresponding training and test sets for the dipeptide features is detected. These results substantiate the observation that the dipeptide-AdaBoost-RF model is robust enough to be designated as the model for prediction of antibody classes for epitope/s in AbCPE server.

### Epitope Prediction Server

**FIGURE 3:**
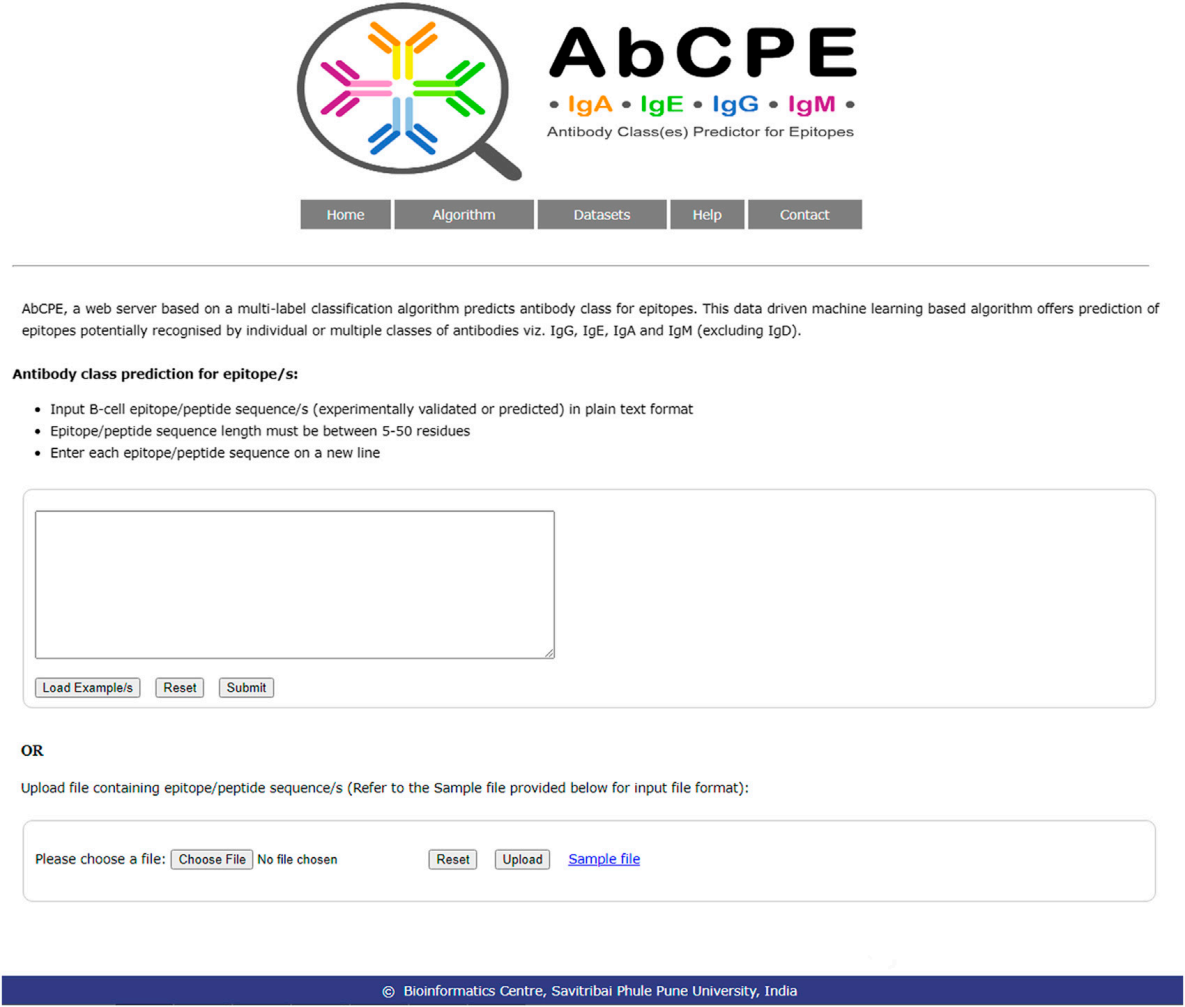
Snapshot of AbCPE home page.

A user-friendly web server entitled AbCPE (http://bioinfo.unipune.ac.in/AbCPE/Home.html) is developed as an implementation of the current multi-label epitope prediction algorithm wherein users can perform predictions and obtain results. A snapshot of home page of AbCPE web server is shown in Figure 3. The dipeptide model based on combination of Binary Relevance, Random Forest and AdaBoost (dipeptide-BR-RF-AdaBoost) has been found to outperform other models on training as well as test dataset. Therefore this model is incorporated in the web server to provide the best results on predictions. To predict the antibody class(es), users need to input epitope sequences that are either predicted by an epitope prediction algorithm or the ones that are characterized experimentally. Therefore, the AbCPE is unique method that not only provides a value addition over the existing layer of B-cell epitope prediction methods but makes the antibody class(es) prediction possible. It thus has the potential to be used as an additional add-on module for rational design of immunotherapeutics.

### Evaluation of SARS-CoV-2 Dataset on Antibody Class(es) Predictor for Epitopes

The prediction efficacy of dipeptide-BR-RF-AdaBoost model integrated in the AbCPE server on test data from SARS-CoV-2 is observed to be very good with Hamming Loss of 0.036 (Table 7). The predictions are especially efficient in case of IgG binding epitopes of novel coronavirus which denotes the major antibody class in the dataset. In case of Precision, Recall and F1 score, the micro averaged values are very high compared to macro averaged values. This is expected since there is high disparity between epitopes for three antibody classes in the dataset. Micro averaged measures are known to be more sensitive to class imbalance in data as compared to macro averaged measures.

**TABLE 7:** Prediction performance of AbCPE server on SARS-CoV-2 data.

Dataset	HL	Micro average			Macro average		
		P	R	F1	P	R	F1
SARS-CoV-2	0.0360	0.9318	0.9265	0.9291	0.2369	0.2460	0.2411

## Discussion

The field of computational B-cell epitope prediction has progressed and evolved at a tremendous pace in recent years with availability of large number of methods which have accelerated the pace of rational design of vaccines. Over the years, majority of these methods have focused on use of different properties of epitopes as well as diverse algorithms to improve accuracy of epitope predictions. However, antibodies display properties like cross-reactivity, polyspecificity and heterospecificity which result in their binding to different epitopes/antigens (Van Regenmortel, 2014). Additionally, studies of antibody specificity have revealed that isotype switching is associated with altered specificity in spite of conservation of V region sequences in antibodies (Janda et al., 2016). This effect has been observed in case of IgG, IgM, IgE and IgA class of antibodies for a variety of antigens. Majority of the existing methods do not account for antibody specificity which has significant effect on recognition of an epitope/antigen. Researchers believe that addressing the B-cell epitope prediction problem from the perspective of antibodies involved in the interactions has the potential to transform the B-cell epitope prediction field (Sela-Culang et al., 2015). Therefore it can be construed that there is a need for development of novel disruptive methods that bring paradigm shift to make epitope predictions relevant to reflect recent knowledge of antigen-antibody recognition as well as demands of synthetic biology. In this work, an attempt has been made to address this complex biological phenomenon through a data driven informatics approach that learns from and incorporates the underlying principles of antigen-antibody recognition with special reference to the immunological systems wherein an epitope is capable of binding to and being recognized by multiple classes of antibodies. The multi-label classification approach has been adopted and implemented for prediction of antibody class(es) for epitopes.

The major hurdle in construction of an efficient epitope prediction tool is associated with availability of epitope sequence data as the quality of datasets used determines the predictive efficiency of the classifier (Greenbaum et al., 2007). Epitope data specific for single class of antibody is available in the IEDB and can be compiled using the IEDB tools. IgD antibody class is an exception in this regard as IgD binding epitope data is not available, which might be due to limited characterization of function. Therefore, IgD is not taken into account while developing AbCPE algorithm. Compilation of the data for epitopes which are able to bind to multiple classes of antibodies is very challenging and special scripts were written to compile and curate this data from the IEDB. Currently the data available for epitopes binding to more than two types of antibodies are less, especially for those involving antibody classes like IgA. Out of the possible 15 combinations of antibody classes to which an epitope can bind, sufficient data were available for 11. The best prediction model obtained in this study provides encouraging performance, especially in view of limited data for some of the label combinations. In the coming years, the efficiency of such types of algorithms is expected to improve further with availability of more and more epitope data for multiple classes of antibodies.

Another observation from compilation of epitope data is the significant variability of the lengths of epitopes. In this study, epitope sequences with their lengths between 5 and 50 amino acids were taken into consideration based on previous reports stating that epitopes with their lengths in this range provide good results (Gupta et al., 2013; Singh et al., 2013).

Evaluation and ranking of the individual features by WEKA-InfoGain facilitated identification of the most informative features from each feature set as well as assessment of significance of individual features. It is generally believed that epitopes are rich in polar and charged amino acids. Results obtained by feature analysis are consistent with this observation. In the four individual features sets, glutamine is observed to be the most critical amino acid with respect to all antibody classes. Analysis of top ranked dipeptide features provides important information on involvement of dipeptides made up of proline with aromatic amino acids like tyrosine and phenylalanine. These dipeptides constitute extremely informative dipeptides in the DC based classifiers such as the one that gives best prediction efficiency and which is subsequently employed in AbCPE server.

Identification of epitopes that are recognized by a single class or multiple classes of antibodies have potential applications in number of different fields, especially in therapeutics and diagnostics. In addition to the previously mentioned role of IgG as blocking antibodies and their usage in developing allergen specific immunotherapy approaches (Aalberse, 2011), IgA antibodies have also found to be potentially important as therapeutic antibodies in allergic diseases (Yamaki and Yoshino, 2015). Monoclonal antibody-based treatments for different diseases have been recognized as one of the foremost approaches in recent times. Research in monoclonal antibody therapeutics has indicated that the immunoglobulin isotype plays an important role in the therapeutic antibody function (Beers et al., 2016). While IgG, especially IgG1 represents the immunoglobulin of choice for the therapeutic usage, other antibody classes have also emerged as promising alternatives. Apart from IgG, other isotypes IgE, IgA and IgM have also shown encouraging results for designing immunotherapeutic approaches in the area of cancer therapeutics (Josephs et al., 2014; Leusen, 2015; Kretschmer et al., 2017).

The capability of an antigen to bind different classes of antibodies can also be utilized to develop more efficient immunodiagnostic methods. This can be illustrated by the earlier discussed example of Dengue virus in which antigen specificity for both IgG and IgM is exploited for rapid and accurate diagnosis of infection (Hapugoda et al., 2007; Lee et al., 2015). The swift and devastating impact of recent coronavirus pandemic has necessitated development of rapid diagnostic approaches. Serological testing has emerged as a very important diagnostic method used increasingly by the clinics. The heterogeneous nature of antibody response after the coronavirus infection leads to generation of antibody isotypes IgG, IgM and IgA which can be used for efficient and early diagnosis of viral infection (Chen et al., 2021). We examined performance of our classifier on SARS-CoV-2 epitope dataset and observed that AbCPE provides effective predictions even though the dataset is highly imbalanced or skewed. As more than 94% examples from this dataset belong to a single antibody class (IgG), the Hamming Loss of 0.036 indicates the ability of the classifier to predict the correct label for this class. Considering the novelty and importance of these epitopes, ability to predict IgG binding epitopes with such proficiency can be very helpful in designing newer diagnostic approaches for novel coronavirus.

To meet the increasing interest and demand for development of novel immunotherapies and immunodiagnostics, next generation disruptive immunoinformatics approaches based on machine learning are envisaged. The choice of machine learning method and classifier therefore becomes an important aspect wherein the decision to opt for binary and/or multi-label classifiers depends on the problem statement. Use of binary classifiers are recommended for prediction of epitopes (from non-epitopes) and prediction of epitopes that bind to a single class of antibody (and not to more than one class) whereas multi-label classifiers are preferred for prediction of epitopes that bind to multiple classes of antibodies, as demonstrated in case of AbCPE. The choice of classifier demands curation of datasets as appropriate for training and testing. The binary classifiers, by definition, require curated positive and negative datasets. Similarly, set of informative attributes/features have been observed to vary for binary and multi-label classifiers and rigorous feature evaluation becomes an essential prerequisite in the process of development of prediction models.

To meet increasing demands to develop diagnostics, therapies and vaccines in the backdrop of emerging and remerging infectious diseases and cancers, the field of immunoinformatics is expected to assist to provide data led exploration of the search space to provide tractable solutions. Therefore we believe that the development of AbCPE, a multi-label prediction server would contribute immensely by narrowing the search space for prediction of epitopes for antibody class/es and thereby demonstrate use of data driven machine learning applications in the field of immunoinformatics.

## Data Availability

Publicly available datasets were analyzed in this study. This data can be found here: http://bioinfo.unipune.ac.in/AbCPE/Dataset.html.
