# Supplementary material for: Antibody Class(es) Predictor for Epitopes (AbCPE): A Multi-Label Classification Algorithm
Source: Front Bioinform. 2021 Sep 7;1:709951. doi: 10.3389/fbinf.2021.709951 (PMC9581038; doi:10.3389/fbinf.2021.709951)
Supplement: Supplementary file 1 [file DataSheet1.docx]

**Supplementary Figures:**


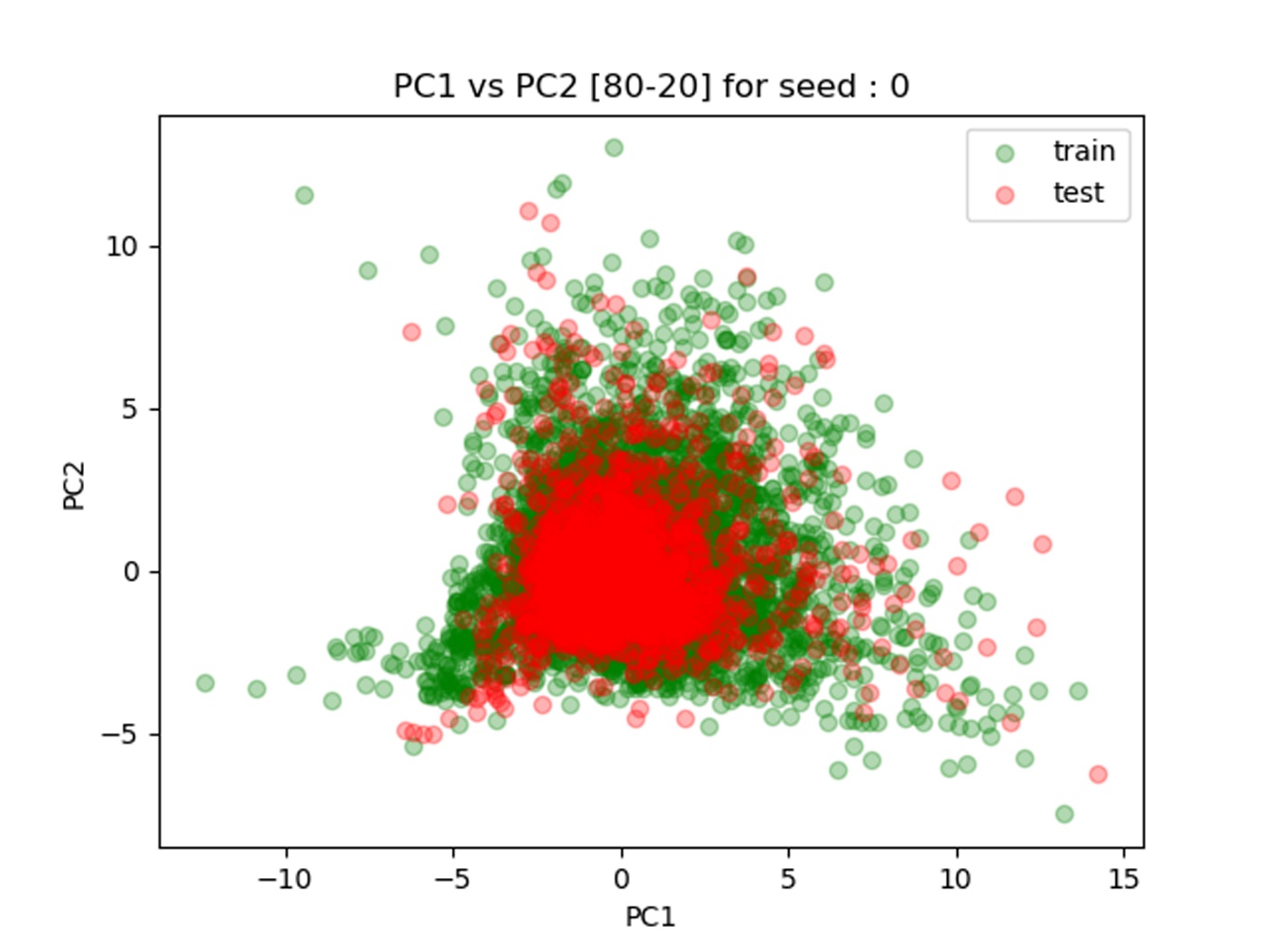


**Supplementary Figure 1.** Principal component analysis (PCA) plot for dipeptide feature set split 1


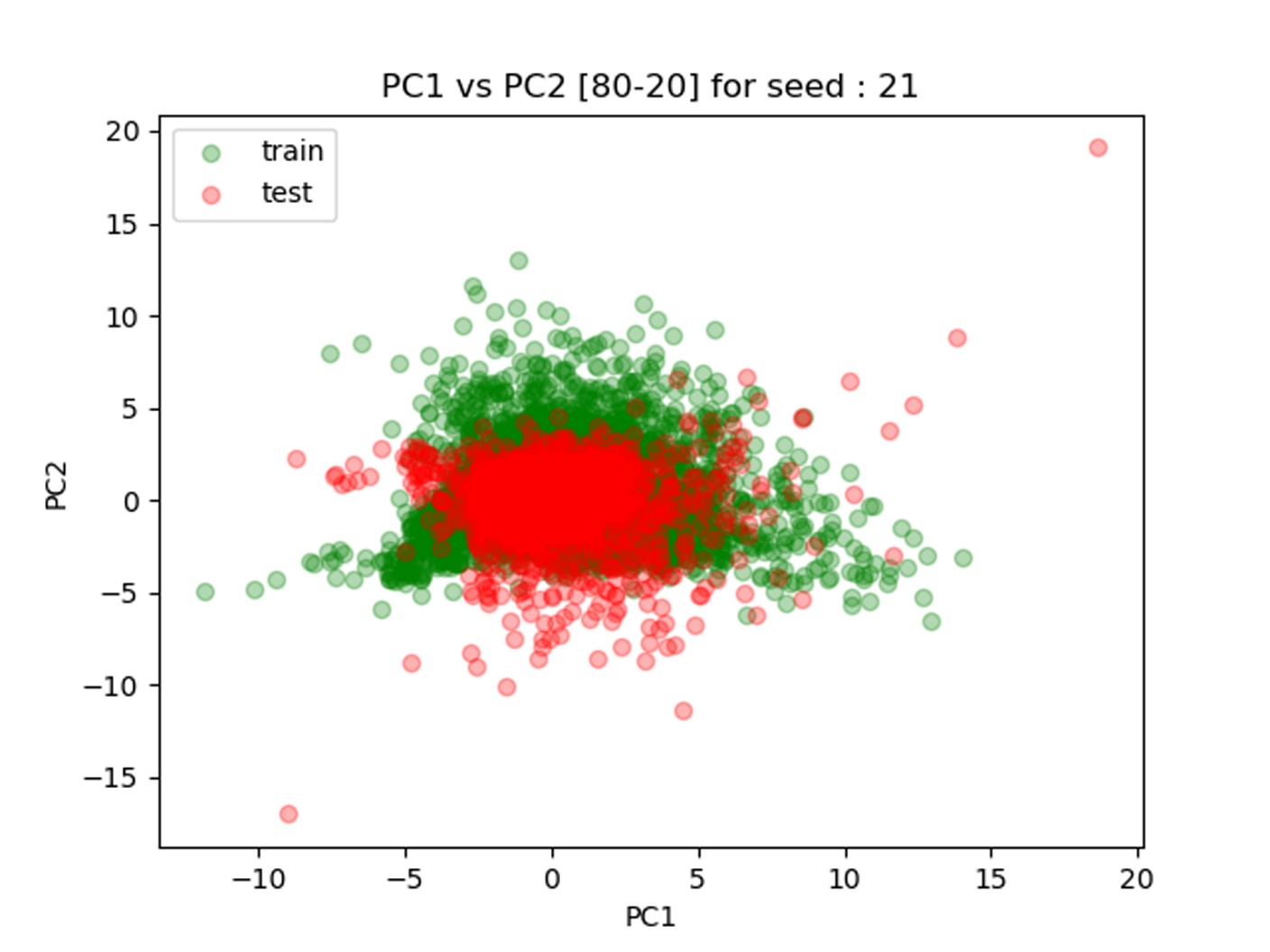


**Supplementary Figure 2.** Principal component analysis (PCA) plot for dipeptide feature set split 2


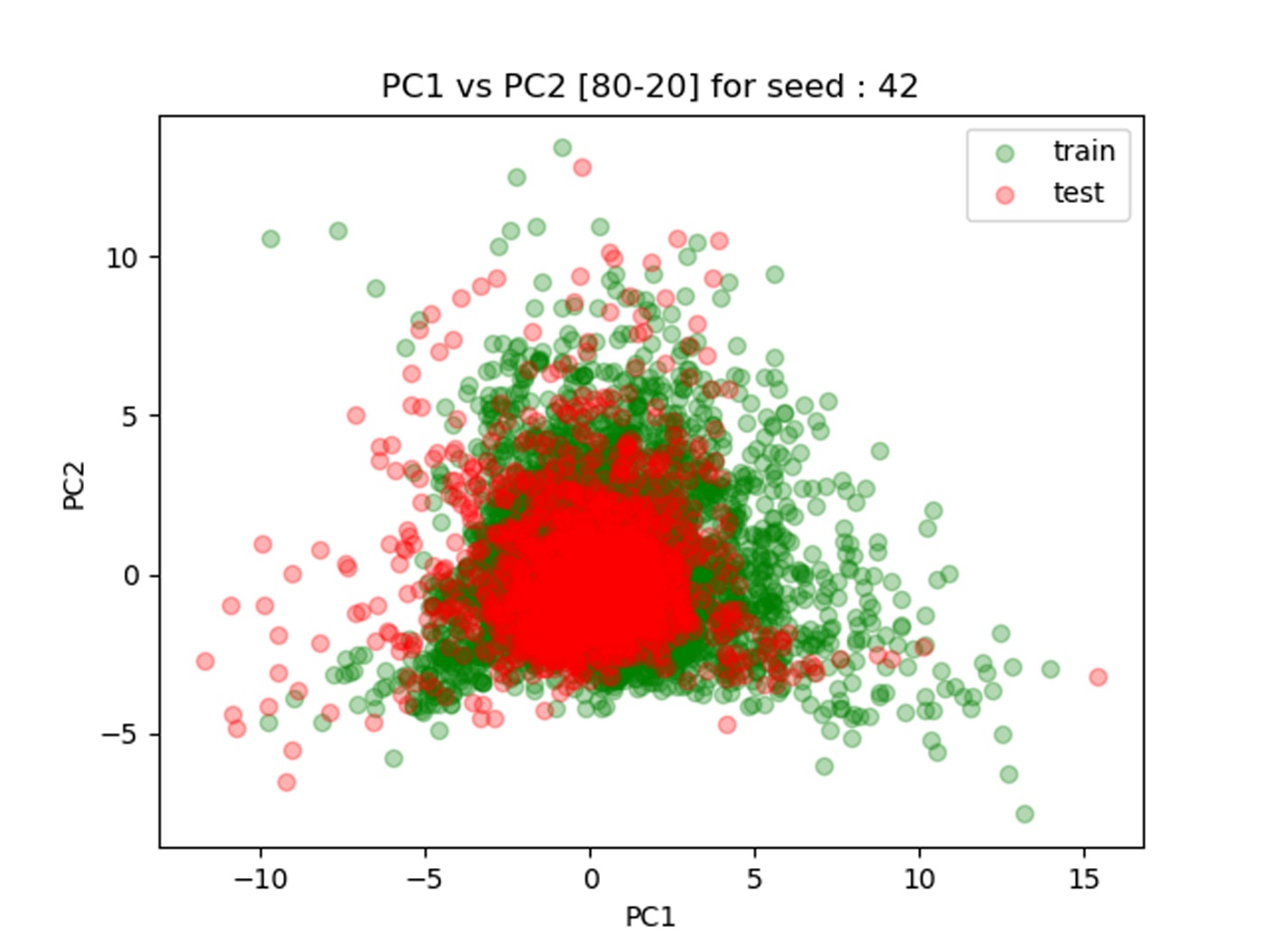


**Supplementary Figure 3.** Principal component analysis (PCA) plot for dipeptide feature set split 3


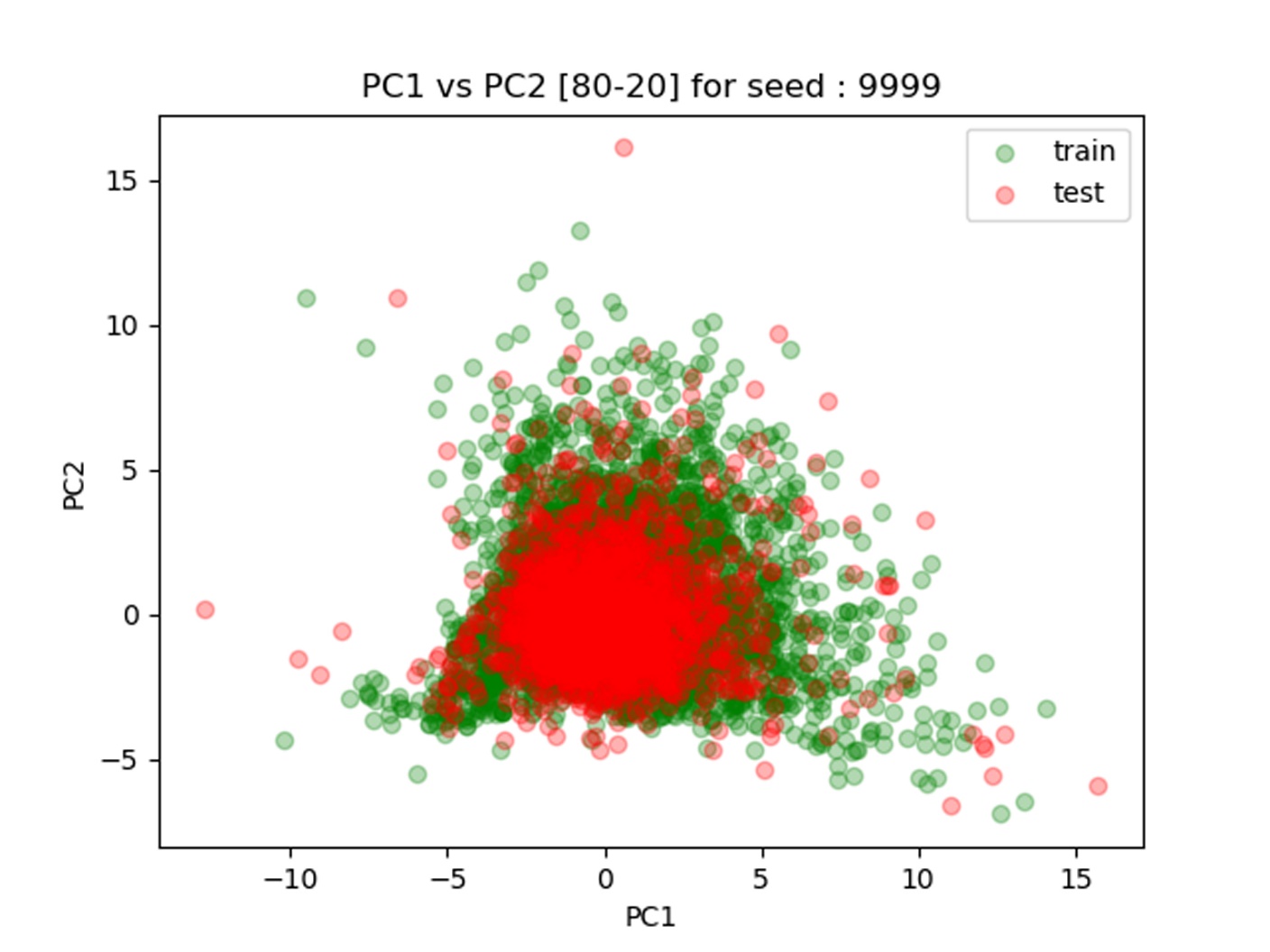


**Supplementary Figure 4.** Principal component analysis (PCA) plot for dipeptide feature set split 4


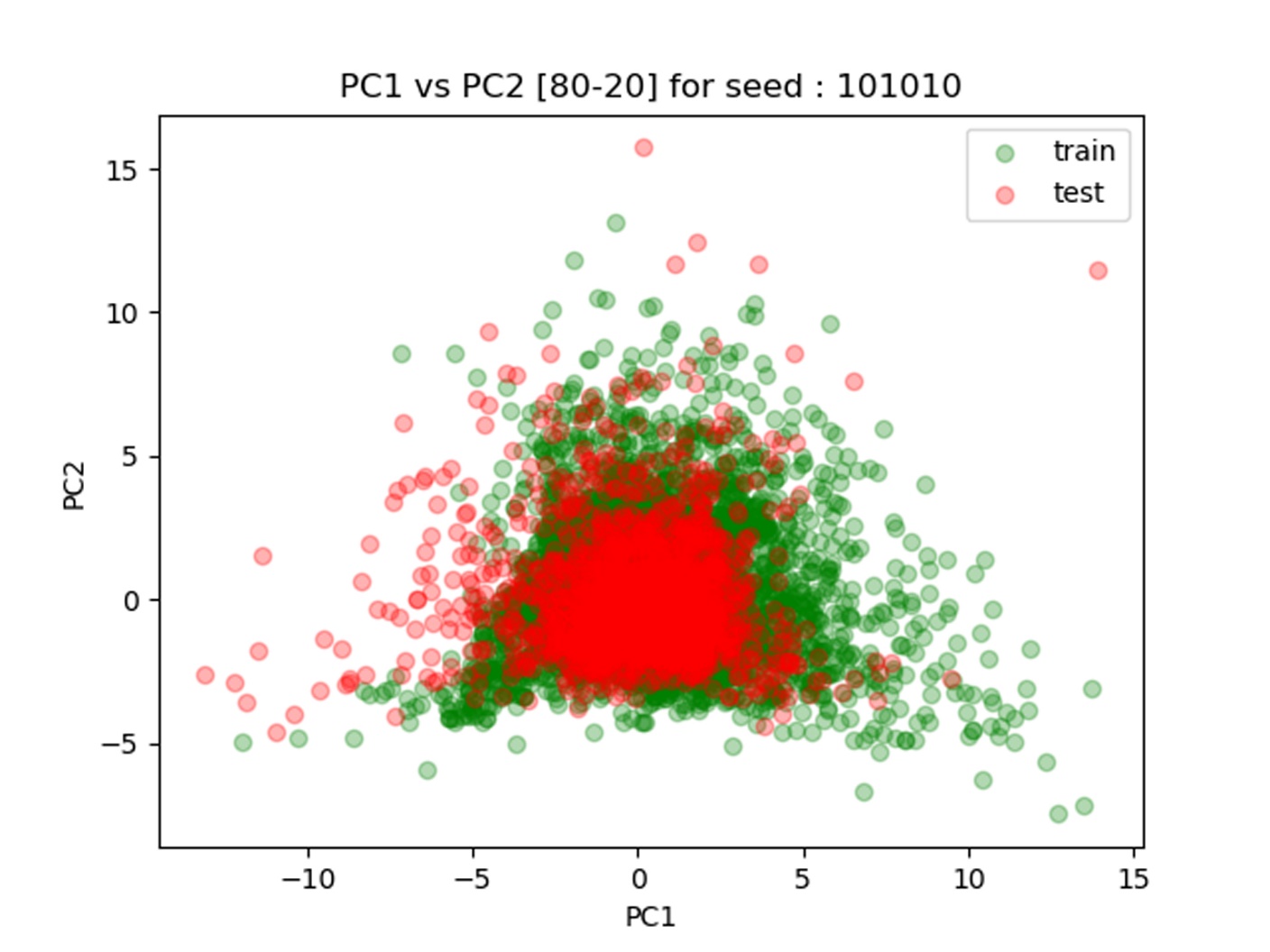


**Supplementary Figure 5.** Principal component analysis (PCA) plot for dipeptide feature set split 5
